# Thermodynamic and Structural Characterization of the Copper(II) Complexes of Peptides Containing Both Histidyl and Aspartyl Residues

**DOI:** 10.1155/2007/30394

**Published:** 2007-12-06

**Authors:** Csilla Kállay, Zoltán Nagy, Katalin Várnagy, Gerasimos Malandrinos, Nick Hadjiliadis, Imre Sóvágó

**Affiliations:** ^1^Department of Inorganic and Analytical Chemistry, University of Debrecen, Debrecen 4010, Hungary; ^2^Department of Colloid and Environmental Chemistry, University of Debrecen, Debrecen 4010, Hungary; ^3^Department of Chemistry, University of Ioannina, Ioannina 45110, Greece

## Abstract

Terminally protected pentapeptides with 2 histidines (Ac-HHVGD-${\text{NH}}_{2}$ and Ac-HVGDH-${\text{NH}}_{2}$) and the terminally free peptides containing both internal aspartyl and C-terminal histidyl residues (FDAH and VIDAH) have been synthesized, and copper(II) complexes studied by potentiometric, UV-Vis, CD, and EPR spectroscopic techniques in solution. Both thermodynamic and spectroscopic data reveal that side chain donor atoms of aspartyl and histidyl residues have a significant contribution to the metal binding affinity of peptide molecules. In the case of terminally protected peptides, the role of the imidazole-N donor functions is reflected in the enhanced stability of the 3N and 4N coordinated copper(II) complexes. The amino and β-carboxylate groups of FDAH and VIDAH create a very effective metal binding site with the (${\text{NH}}_{2}$, ${\text{N}}^{-}$, $\beta \text{-}{\text{COO}}^{-}$) and (${\text{NH}}_{2}$, ${\text{N}}^{-}$, ${\text{N}}^{-}$, $\beta \text{-}{\text{COO}}^{-}$) coordination modes including the N-termini, while the histidine sites are available for the formation of the (${\text{N}}_{\text{im}}$, ${\text{N}}^{-}$, ${\text{N}}^{-}$) binding mode resulting in the preference of dinuclear complex formation.

## 1. INTRODUCTION

Metal complexes of peptides are promising and frequently used models of the active centres of various metalloenzymes. The high metal binding affinity of peptide ligands generally requires the presence of strongly coordinating side chain residues including the carboxylate and imidazole functions of aspartyl and histidyl residues, respectively. The metal ion speciation of these systems and the structural parameters of the predominating species, however, significantly depend on the amino acid sequences of the peptides. The most common features of the coordination chemistry of peptides have already been reviewed by several authors [1, 2] and the governing roles of histidyl and aspartyl or glutamyl residues have also been discussed recently [3, 4]. A great number of studies revealed the enhanced stability of peptide complexes containing the histidine amino acid close to the terminal amino groups, for example, the Xaa-His-Xaa-Xaa and Xaa-Xaa-His-Xaa sequences [3, 4]. In the case of the terminally protected peptides, the anchoring role of internal histidyl residues has been well documented and various forms of amide-bonded complexes have been characterized [5–8]. More recently, the copper(II) complexes of multihistidine peptides have also been investigated and the formation of macrochelates suggested [9–11]. The peptide fragments of the hormone thymopoietin served as the simplest models to study the effect of side chain carboxylate functions on the complex formation processes of peptides [12, 13]. It was found that the presence of β-carboxylate functions of aspartyl residues always results in the enhanced thermodynamic stability of peptide complexes and in the case of the Xaa-Xaa-Asp-Xaa sequences the coordination of β-carboxylate-O donor is able to prevent the deprotonation of subsequent amide functions. A systematic study on tetrapeptides containing more aspartyl residues came to similar conclusions [14]. The metal binding abilities of the peptides of aspartyl and glutamyl residues were compared recently and the stabilizing role of β-carboxylate functions of aspartyl residues was demonstrated over that of the γ-carboxylate groups of glutamic acid [15]. The versatility of the complex formation processes of aspartyl and glutamyl peptides can be further enhanced by the involvement of the side chain carboxylates in amide binding. The terminal and amide donor functions are separated in these peptides and it generally results in very complicated solution equilibria including the formation of polynuclear species [16, 17]. The complexity of these systems is best exemplified by the metal ion interactions of glutathione [18]. On the other hand, only a few papers have been published on the complex formation processes of peptides containing both histidyl and aspartyl residues. The tetrapeptide HisValGlyAsp corresponds to the 78–81 amino acid fragment of the zinc binding site of the enzyme CuZn-SOD [19] and its copper(II) and zinc(II) complexes were studied by potentiometric and spectroscopic methods both for the free and terminally protected peptides. The data revealed the governing role of histidyl residue in the complexation processes with a slight stability enhancement from the aspartyl moiety [20–22]. The active centre of the enzyme, however, contains more than one histidyl residues, while the effect of aspartic acid is more pronounced if it is located in internal position. Now in this paper we report the synthesis and the results obtained for the copper(II) complexes of four new peptide ligands: (a) the terminally protected pentapeptides including one aspartyl and two histidyl moieties, Ac-HisHisValGlyAsp-NH_2_ (Ac-HHVGD-NH_2_), Ac-HisValGlyAspHis-NH_2_ (Ac-HVGDH-NH_2_), and (b) the terminally free peptides containing internal aspartyl and C-terminal histidyl residues, PheAspAlaHis (FDAH) and ValIleAspAlaHis (VIDAH).

## 2. EXPERIMENTAL

### 2.1. Materials

The peptides Ac-HisHisValGlyAsp-NH_2_ (abbreviated as Ac-HHVGD-NH_2_), Ac-HisValGlyAspHis-NH_2_ (Ac-HVGDH-NH_2_), PheAspAlaHis (FDAH), and ValIleAspAlaHis (VIDAH) were synthesized in the solid state using 2-chlorotrityl chloride resin and its modified form (H-Linker-CLTR resin). All solvents and chemicals used for synthesis were from commercial sources in the highest available purity and used without further purification. The resins and the protected amino acids (Fmoc–His(${\text{N}}_{\text{im}}$–Mtt)–OH, Fmoc–Val–OH, Fmoc–Gly–OH, Fmoc-Asp(OtBu)-OH, Fmoc–Phe–OH, Fmoc–Ala–OH, and Fmoc–Ile–OH were purchased from CBL Chemical Ltd., (Patras, Greece) and details of the peptide synthesis have already been described [20].

A stock solution of copper(II) chloride was prepared from analytical grade reagent and the concentration was checked gravimetrically via the precipitation of oxinate.

### 2.2. Potentiometric measurements

The pH-potentiometric titrations in the pH range 2.5–11.5 were performed in 3 cm^3^ samples in the concentration range $2\times 10^{-3}$–$8\times 10^{-3}$ mol dm^−3^ at 1 : 2, 1 : 1, and 2 : 1 metal ion to ligand ratios. The measurements were made with a MOLSPIN pH meter equipped with a 6.0234.100 combined electrode (Metrohm) and a MOL-ACS microburette controlled by computer.

The titrations were performed with carbonate-free stock solution of potassium hydroxide of known concentration. During the titration argon was bubbled through the samples to ensure the absence of oxygen and carbon dioxide and for stirring of the solutions. All pH-potentiometric measurements were carried out at a constant ionic strength of 0.2 M KCl and at a constant temperature (298 K). The number of experimental points reached around 50 to 70 data (cm^3^-pH) for each titration curve. The pH readings were converted into hydrogen ion concentration as described earlier [23]. Protonation constants of the ligands and the overall stability constants ($\log\beta_{\text{pqr}}$) of the complexes were calculated by means of general computational programs, PSEQUAD [24]and SUPERQUAD [25] using (1) as follows

(1) $$p\text{M}+q\text{H}+r\text{L}⇌{\text{M}}_{p}{\text{H}}_{q}{\text{L}}_{r},\;\beta_{\text{pqr}}=\frac{\left[M_{\text{p}}H_{\text{q}}L_{\text{r}}\right]}{{\left[M\right]}^{\text{p}}{\left[H\right]}^{\text{q}}{\left[L\right]}^{\text{r}}}.$$

### 2.3. Spectroscopic studies

UV-Vis spectra of the copper(II) complexes were recorded on a Hewlett Packard HP 8453 diode array and on a Perkin-Elmer Lambda 25 double beam spectrophotometer in the same concentration range as used for pH-potentiometry. The EPR continuous wave spectra were recorded at 120 K in a Varian E109 spectrometer operated at the X-band and equipped with a cryostat of Oxford Instruments. Experimental conditions: microwave power 20 mW, modulation amplitude 10 G, and sweep width 2000 G. The concentration of copper(II) and peptides were the same as used for pH-potentiometry.

CD spectra of copper(II) complexes were recorded on a JASCO J-81 spectropolarimeter using 1 or 10 mm cells in the 200–800 nm range in the same concentration range as used for potentiometry. CD spectra of the individual species were calculated by the same general program (PSEQUAD) as used for the evaluation of potentiometric measurements.

## 3. RESULTS AND DISCUSSION

### 3.1. Protonation constants of the ligands

Protonation macroconstants of the ligands have been determined by pH-potentiometric measurements and the equilibrium data are collected in Table 1.

It is clear from Table 1 that the peptides have 3 or 4 protonation sites which can be assigned to the terminal amino and carboxylate groups and the side chain imidazole and β-carboxylate functions. In agreement with the common oligopeptides the terminal amino groups are the most basic sites of the molecules, while the C-terminal α-carboxylic groups are the most acidic. Taking into account the small differences of the stepwise pK values, the unambiguous assignment of the protonation sites would require the determination of microscopic protonation constants. In the case of the terminally protected peptides these values have already been reported [26] and the data revealed the complete overlap in the basicity of the two imidazole moieties.

### 3.2. Copper(II) complexes of the terminally protected peptides (Ac-HHVGD-NH_2_ and Ac-HVGDH-NH_2_)

The stability constants of the copper(II) complexes of all peptides were determined by potentiometric measurements and the data are collected in Table 2. The terminal amino groups of peptides are the most common anchors for metal binding and, as a consequence, the complex formation processes of the terminally free and protected peptides are significantly different and the results are discussed in separate subsections.

**Figure 1:**
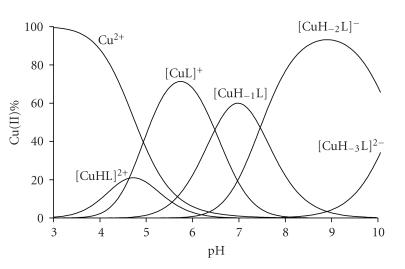
Metal ion speciation of the copper(II)-Ac-HHVGD-NH_2_ system at 1 : 1 ratio (${\text{c}}_{\text{L}}=2\cdot 10^{-3}$ mol/dm^3^).

It is clear from Table 2 that the complex formation processes of the two terminally protected peptides Ac-HHVGD-NH_2_ and Ac-HVGDH-NH_2_ are very similar to each other and much simpler than those of the free peptides. The speciation is shown for the copper(II)-Ac-HHVGD-NH_2_ system in Figure 1, but a very similar speciation can be drawn also for Ac-HVGDH-NH_2_. Figure 1 clearly demonstrates that complex formation starts in a slightly acidic pH range, above pH 3.5 when only the imidazole functions can be protonated. Thus, the species [CuHL] and [CuL] can be characterized by the binding of imidazole-N donor atoms, in the forms of monodentate imidazole binding $\left({\text{N}}_{\text{im}}\right)$ and macrochelate $\left(2\times {\text{N}}_{\text{im}}\right)$, respectively. The stability constants calculated for the Cu-${\text{N}}_{\text{im}}$ binding mode [log K(Cu + HL) values in Table 2] are slightly higher than those of simple monohistidine peptides [8] suggesting that the *β*-carboxylate functions of aspartyl residues also have a slight contribution to metal binding. The data obtained for the macrochelates show similar tendencies if they are compared to those of the copper(II) complexes of the corresponding bis-histidine peptides [11, 22].

The spectroscopic parameters of the major species are collected in Table 3 and support the above-mentioned conclusions. In the case of the species [CuL] both the absorption maxima and EPR parameters agree well with those of 2N coordinated complexes in a slightly distorted environment. No measurable CD spectra can be recorded in these systems below pH 5 and it provides an unambiguous proof for the exclusive binding of imidazole-N donor atoms in the species [CuHL] and [CuL]. On the other hand, characteristic CD spectra can be recorded in parallel with the formation of the [$\text{Cu}{\text{H}}_{-n}\text{L}$] species supporting that the extra deprotonation reactions come from the metal ion coordination of the deprotonated amide functions and mixed hydroxo complexes are not present in measurable concentration.

**Scheme 1:**
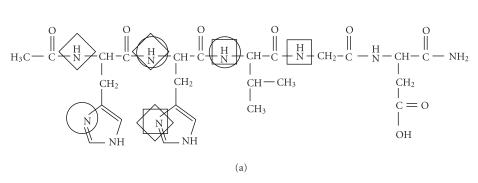

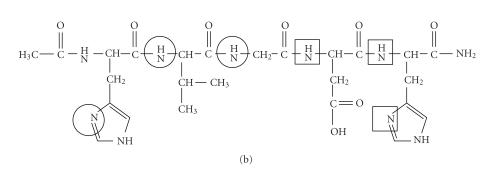


The metal binding sites of the ligands in the species [CuH_−n_L] can be described by the $\left[{\text{N}}_{\text{im}},n\times {\text{N}}^{-}\right]$ coordination modes (n = 1–3) containing increasing number of amide nitrogen donor atoms. The pK(n/n−1) values reveal that the deprotonation of amide functions takes place in consecutive reactions while the cooperative deprotonation of the first two amide functions was characteristic of many other histidine containing peptides [8]. However, in the case of these ligands only one of the imidazole residues takes part in the formation of chelate ring with the amide function and the other histidine can occupy the free coordination sites creating a macrochelate structure which stabilizes the species [CuH_−1_L]. The spectroscopic data strongly support that the macrochelate is present in the [CuH_−2_L] species, too. The comparison of UV-Vis and EPR parameters in Table 3 with those of the $\left({\text{N}}_{\text{im}},2\times {\text{N}}^{-}\right)$ coordinated complexes of prion peptide fragments containing only one histidyl residues [8, 27] reveals the increase in the number of coordinated nitrogen donors suggesting th$\left({\text{N}}_{\text{im}},2\times {\text{N}}^{-},{\text{N}}_{\text{im}}\right)$ binding mode for the [CuH_−2_L] species of Ac-HHVGD-NH_2_ and Ac-HVGDH-NH_2_. This coordination mode suppresses the deprotonation of the third amide functions to a more alkaline pH range (pH *>* 9) than it was reported for other histidine peptides (pH *<* 9). The pK(−2/−3) value is especially high for the ligand Ac-HHVGD-NH_2_, but in this case, starting from the histidyl residues, the amide functions can be deprotonated only towards the C-terminus in the form of (7,5,5)-membered fused chelates while they are (6,5,5)-membered ones for the other ligands. It is also important to note that various coordination isomers of the species [CuH_−1_L] and [CuH_−2_L] can exist because there are several possibilities for the deprotonation of the first two amide functions of both ligands. It is demonstrated by Scheme 1 where the metal binding sites of the various coordination isomers are indicated by different squares and circles. The results of our previous studies [11] on the copper(II) complexes of multihistidine peptides revealed that the spectroscopic parameters of these coordination isomers are very similar to each other. As a consequence, we cannot estimate the ratio of the various isomers in solution, but previous studies definitely support that the isomers built up from 6- and 5-membered chelates predominate over the 7-membered ones.

In the previous paragraph the equilibrium and spectroscopic data reported for the species [CuHL] and [CuL] revealed the stabilization role of β-carboxylate function in the Cu–${\text{N}}_{\text{im}}$ binding mode. On the contrary, the comparison of the recent data obtained for the species [Cu${\text{H}}_{-\text{n}}$L] with those reported for peptides without additional carboxylate functions suggests that *β*-carboxylate groups of aspartyl residues do not have a significant contribution to the overall stability and structural parameters of amide-bonded complexes.

### 3.3. Copper(II) complexes of the terminally free peptides (FDAH and VIDAH)

**Figure 2:**
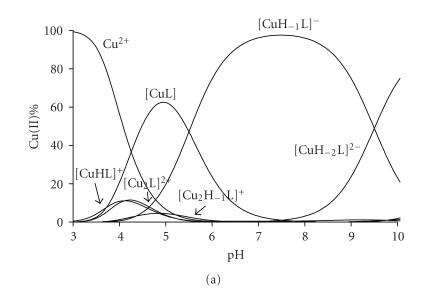

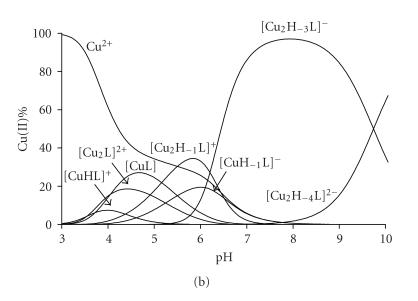
Metal ion speciation of the copper(II)-FDAH system in (a) equimolar samples and (b) at 2 : 1 metal to ligand ratio, (${\text{c}}_{\text{L}}=2\cdot 10^{-3}$ mol/dm^3^).

The metal ion speciation of the copper(II)-FDAH system is shown in Figure 2 at two different metal ion to ligand ratios. It is clear from Figure 2 and Table 2 that both mono- and di-nuclear complexes are formed in this system, thus the peptide is able to keep more than one equivalent of copper(II) ion in solution even under alkaline conditions.

The complexation starts in slightly acidic solution below pH 4 with the formation of the protonated complex [CuHL]. The amino groups are generally the primary ligating sites of the non-protected peptides [1, 2], thus the amino and neighbouring carbonyl groups are bonded in this species, while the imidazolium group remains protonated. The concentration of [CuHL] is, however, very low at any metal ion to ligand ratios and the exact spectral parameters cannot be determined. The formation of the species [CuL] is accompanied with the appearance of detectable CD extrema (see Table 4 for spectral parameters) suggesting that formation of [CuL] from [CuHL] is not simply the deprotonation of the imidazolium group, but it is the deprotonation of the first amide function and the real stoichiometry of [CuL] is [Cu(H_−1_L)H]. The outstanding stability of the (NH_2_, N^−^,β-COO^−^) coordination mode has already been reported in several other peptides and the stability constant of such a species is around 2 log⁡ units [14]. Correcting the $\log\beta_{101}$ value with the pK of imidazole of FDAH log⁡ K = (9.23−6.89=) 2.34 can be calculated for [Cu(H_−1_L)H] supporting the above-mentioned coordination mode. It is also a common feature of the peptides with Xaa-Asp-Xaa sequences, that the deprotonation of the second amide functions is significantly suppressed as compared to those of the common oligopeptides. Namely, the pK(–1/–2) = 9.64 was reported for the tetrapeptide ADAA [14], which is in a very good agreement with our data in Table 2. As a consequence, the species [CuH_−1_L] will predominate in a wide pH range including the physiological values. Its stability constant is, however, higher than the characteristic 2 log⁡ units, because the deprotonated side chain imidazole can occupy the fourth coordination site around the metal ion. This assumption is supported by the slight blue shift of the absorption maxima upon the formation of [CuH_−1_L] from [CuL].

**Scheme 2:**
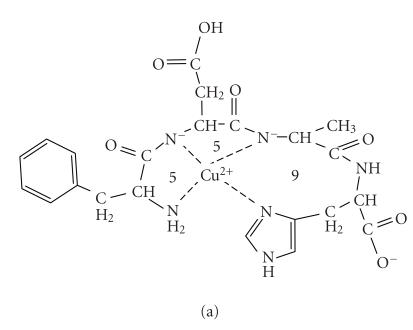

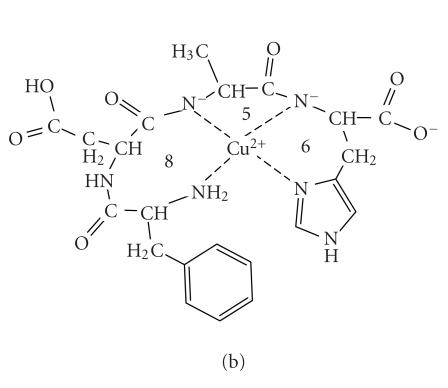


A further base consuming process is detected above pH 9, when both amino and imidazole nitrogen donors are coordinated. The significant blue shift of the absorption maxima and characteristic changes of CD spectra unambiguously prove the coordination of the second amide nitrogens, but in the forms of various coordination isomers. These isomers can include the (NH_2_, N^−^, N^−^, ${\text{N}}_{\text{im}}$) coordination mode starting from the N-terminus (see Scheme 2(a)) and the (${\text{N}}_{\text{im}}$, N^ −^, N^−^, (NH_2_)) coordination mode starting from the C-terminal histidyl residues (see Scheme 2(b)). The determination of the exact ratios of the isomers would require the study of the copper(II) complexes of some model peptides, but the comparison of the CD spectra of copper(II)-FDAH with those reported for other histidine peptides [28] suggests that the metal binding from the histidyl site may predominate over the amino site in alkaline solutions. It means that the coordination of the second amide function is not simply a deprotonation reaction but it is accompanied with the rearrangement of the metal binding sites of the peptides. Namely, the terminal amino group and the subsequent amide are the primary ligating sites below pH 9, but they are partly replaced by the imidazole and preceding amide functions at high pH values. In agreement with this assumption the deprotonation of the third amide function (pK(−2/−3) values) is very much suppressed as compared to that of ADAA (= 9.88).

**Scheme 3:**
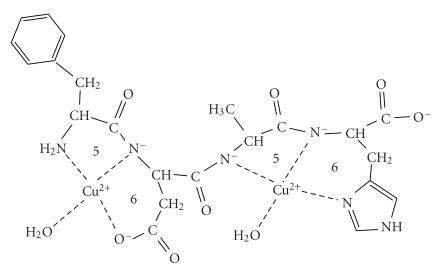


Figure 2(b) makes it clear that the metal binding of the histidyl residue is facilitated in the presence of excess metal ions. The major dinuclear species at 2 : 1 metal ion to ligand ratios is [Cu_2_H_−3_L] which can be easily built up from the superposition of the N-terminal and histidyl binding sites (see Scheme 3). All three amide functions take part in metal binding in this species and both copper(II) ions have one free coordination site. As a consequence, the formation of mixed hydroxo complexes is possible by increasing pH, but their formation is accompanied with very small spectral changes.

**Figure 3:**
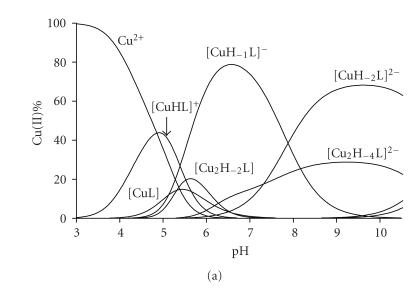

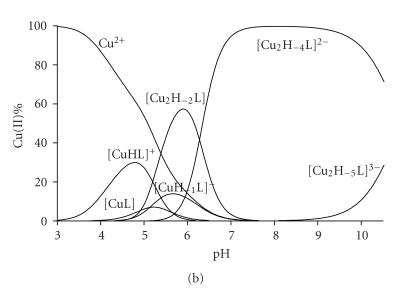
Metal ion speciation of the copper(II)-VIDAH system in (a) equimolar samples and (b) at 2 : 1 metal to ligand ratio, (${\text{c}}_{\text{L}}=2\cdot 10^{-3}$ mol/dm^3^).

**Figure 4:**
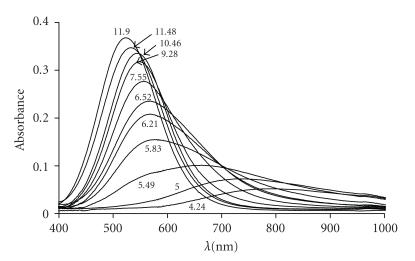
Visible spectra of the copper(II)-VIDAH system in equimolar samples at different pH values (${\text{c}}_{\text{L}}=2\cdot 10^{-3}$ mol/dm^3^).

**Figure 5:**
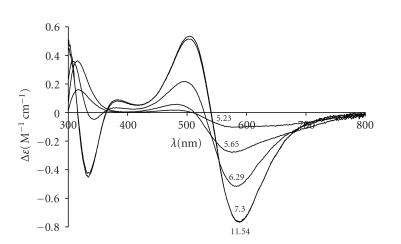
CD spectra of the copper(II)-VIDAH system at 2 : 1 metal ion to ligand ratios at different pH values (${\text{c}}_{\text{L}}=2\cdot 10^{-3}$ mol/dm^3^).

The metal ion speciation of the copper(II)-VIDAH system is shown in Figure 3 at 1 : 1 and 2 : 1 metal ion to ligand ratios. The comparison of Figures 2 and 3 reveals a high similarity in the complex formation processes of FDAH and VIDAH, but some important differences can also be observed. The stoichiometry of the first species formed below pH 5 is [CuHL] and taking into account the pH values it can contain only the imidazole-N atom in protonated from. Its concentration is, however, significantly higher than its counterpart formed in the copper(II)-FDAH system, suggesting that the contribution of β-carboxylate group to metal binding is enhanced with the pentapeptide. The concentration of [CuL] is negligible and the species [CuH_−1_L] predominates in the pH range from 5.5 to 7.5. The coordination mode of the [CuH_−1_L] species of N-terminally free peptide complexes is generally described by the formation of 2N complexes (NH_2_,N^−^), but the spectroscopic data (see Table 4) contradict with this assumption. Both absorption and CD spectra support the metal binding of 3 nitrogen donor atoms, that is, the existence of (NH_2_, N^−^, N^−^, β-COO^−^) coordination. The outstanding stability of this binding mode has already been discussed in the introduction and the spectral parameters in Table 4 are also in good agreement with literature data [12, 14]. As a consequence, the real stoichiometry of [CuH_−1_L] is [Cu(H_−2_L)H] containing two deprotonated amide groups, while the imidazolium group remains protonated. The corresponding pK(–1/–2) value is higher than that of the free ligand, but similar decrease in the acidity of non-coordinating side chains has already been reported in some other peptide complexes containing coordinated amide functions [29, 30]. The small blue shift of the absorption spectra (see Figure 4) partly contradicts with the assumption that only the deprotonation of the side chain imidazolium group occurs in the pH range 6.5 to 9.5, but the speciation curves in Figure 3(a) clearly show that formation of [CuH_−2_L] significantly overlap with the formation of dinuclear species affecting the overall spectra of the samples. At the first sight it is surprising that the concentration of the dinuclear complex is more than 30% in equimolar samples, but the application of the statistical treatment for the distribution of metal ions among equivalent binding sites results in a ratio of 25% for dinuclear species [31]. Figure 3(b) demonstrates that the major dinuclear species [Cu_2_H_−4_L] predominates in a very wide pH range (pH 7–10) and its exclusive formation is further supported by the CD spectra depicted at Figure 5.

**Scheme 4:**
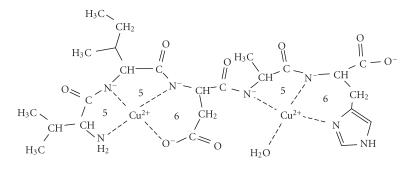


The metal binding sites of [Cu_2_H_−4_L] (see Scheme 4) can be easily obtained from the superposition of the (NH_2_, N^−^, N^−^ β-COO^−^) and $\left({\text{N}}_{\text{im}},2\times {\text{N}}^{-}\right)$ coordination modes starting from the N- and C-termini of the peptide, respectively. This species has only one free coordination site which is available for hydroxo complex formation in strongly alkaline samples.

## 4. CONCLUSIONS

Both thermodynamic and spectroscopic data provide further evidence that side chain donor atoms of aspartyl and histidyl residues have a significant influence on the metal binding affinity of peptide molecules. In the case of terminally protected peptides, like Ac-HHVGD-NH_2_ and Ac-HVGDH-NH_2_ the role of the imidazole-N donor functions are especially striking resulting in the formation 3N and 4N coordinated copper(II) complexes. The β-carboxylate functions of aspartic acid is much less pronounced affecting only the stability constants of the Cu–${\text{N}}_{\text{im}}$-bonded species. The presence of free terminal amino group, however, significantly changes the complex formation processes. The amino and β-carboxylate groups of FDAH and VIDAH create a very effective metal binding site with the (NH_2_, N^−^, β-COO^−^) and (NH_2_, N^−^, N^−^, β-COO^−^) coordination modes, respectively, while the histidine sites are available for the formation of the (${\text{N}}_{\text{im}}$, N^−^, N^−^) binding mode. As a consequence, dinuclear species are formed in high concentration with these peptides, while the mononuclear species may have a series of coordination isomers in solution.

## Figures and Tables

**Table 1:** Protonation macroconstants and stepwise pK values of the peptides T = 298 K, I = 0.2 mol/dm^3^ KCl (charges are omitted).

Species	Ac-HHVGD-NH_2_	Ac-HVGDH-NH_2_	FDAH	VIDAH
[HL]	6.91(1)	6.79(1)	7.67(1)	7.84(1)
$\left[{\text{H}}_{2}\text{L}\right]$	12.97(1)	12.72(1)	14.56(1)	14.74(1)
$\left[{\text{H}}_{3}\text{L}\right]$	16.40(2)	16.34(2)	18.31(1)	18.60(2)
$\left[{\text{H}}_{4}\text{L}\right]$	—	—	21.11(2)	20.95(3)
pK(amino)	—	—	7.67	7.84
pK(${\text{N}}_{\text{im}}$)	6.91	6.79	6.89	6.90
pK(${\text{N}}_{\text{im}}$)	6.06	5.93	—	—
pK(β-COO^−^)	3.43	3.62	3.75	3.86
pK(α-COO^−^)	—	—	2.80	2.35

**Table 2:** Stability constants ($\log\beta_{\text{pqr}}$) of the copper(II) complexes of peptides T = 298 K, I = 0.2 mol/dm^3^ KCl (charges are omitted).

Species	Ac-HHVGD-NH_2_	Ac-HVGDH-NH_2_	FDAH	VIDAH
$\left[{\text{Cu}}_{}\text{H}\text{L}\right]$	10.87(4)	10.62(9)	12.95(6)	12.98(2)
$\left[{\text{Cu}}_{}\text{L}\right]$	6.24(2)	6.42(2)	9.23(1)	7.33(6)
$\left[{\text{CuH}}_{-1}\text{L}\right]$	−0.24(3)	−0.36(4)	3.73(1)	2.20(2)
$\left[{\text{CuH}}_{-2}\text{L}\right]$	−7.70(3)	−7.57(3)	−5.77(2)	−5.68(5)
$\left[{\text{CuH}}_{-3}\text{L}\right]$	−18.04(3)	−17.03(4)	−17.36(2)	−17.27(5)
$\left[{\text{Cu}}_{2}\text{L}\right]$	—	—	11.56(8)	—
$\left[{\text{Cu}}_{2}{\text{H}}_{-1}\text{L}\right]$	—	—	6.65(7)	—
$\left[{\text{Cu}}_{2}{\text{H}}_{-2}\text{L}\right]$	—	—	—	−0.16(1)
$\left[{\text{Cu}}_{2}{\text{H}}_{-3}\text{L}\right]$	—	—	−5.76(3)	—
$\left[{\text{Cu}}_{2}{\text{H}}_{-4}\text{L}\right]$	—	—	−15.50(5)	−12.71(3)
$\left[{\text{Cu}}_{2}{\text{H}}_{-5}\text{L}\right]$	—	—	—	−23.63(5)
pK(1/0)	4.63	4.20	3.62	5.65
pK(0/−1)	6.48	6.78	5.50	5.13
pK(−1/−2)	7.46	7.21	9.50	7.88
pK(–2/–3)	10.34	9.46	11.59	11.59
log⁡K(Cu+HL)	3.96	3.83	—	—

**Table 3:** Spectroscopic parameters of the major species formed in the copper(II)-Ac-HHVGD-NH_2_ and Ac-HVGDH-NH_2_ systems.

Ligand	Species	Vis $\left(\lambda_{\max}/\varepsilon \right)$ (nm/dm^3^mol^−1^cm^−1^)	EPR (g_||_/A_||_) (−/10^−4^cm^−1^)	CD $\left(\lambda_{\max}/\Delta \varepsilon \right)$ (nm/dm^3^mol^−1^cm^−1^)
Ac-HHVGD-NH_2_	[CuHL]	770/55	2.328/153	—
	[CuL]	685/46	2.315/153	—
	$\left[{\text{CuH}}_{-1}\text{L}\right]$	635/61	2.267/177	650/+0.23
				330/–0.26
	$\left[{\text{CuH}}_{-2}\text{L}\right]$	555/109	2.205/179	720/+0.21
				590/–0.47
				500/+0.30
				360/–0.78
	$\left[{\text{CuH}}_{-3}\text{L}\right]$	545/121	2.190/195	580/–0.28
				350/–0.47

Ac-HVGDH-NH_2_	[CuL]	680/64	2.315/155	—
	$\left[{\text{CuH}}_{-1}\text{L}\right]$	625/73	2.285/163	—
	$\left[{\text{CuH}}_{-2}\text{L}\right]$	585/81	2.232/188	630/+0.76
				500/+0.13
	$\left[{\text{CuH}}_{-3}\text{L}\right]$	555/121	2.195/198	600/–0.83
				500/+0.79
				310/–1.43

**Table 4:** Spectroscopic parameters of the major species formed in the copper(II)-FDAH and VIDAH systems.

Ligand	Species	Vis $\left(\lambda_{\max}/\varepsilon \right)$ (nm/dm^3^mol^−1^cm^−1^)	CD $\left(\lambda_{\max}/\Delta \varepsilon \right)$ (nm/dm^3^mol^−1^cm^−1^)
FDAH	[CuL]	635/80	630/+0.20
	$\left[{\text{CuH}}_{-1}\text{L}\right]$	590/90	595/+0.35
	$\left[{\text{CuH}}_{-2}\text{L}\right]$	560/135	580/–0.50
			500/+0.30
			325/+1.55
	$\left[{\text{CuH}}_{-3}\text{L}\right]$	545/130	540/–0.75
			320/+0.85
	$\left[{\text{Cu}}_{2}{\text{H}}_{-3}\text{L}\right]$	600/130	605/–0.70
			515/+0.60
	$\left[{\text{Cu}}_{2}{\text{H}}_{-4}\text{L}\right]$	565/125	605/–0.70
			515/+0.65

VIDAH	$\left[{\text{CuH}}_{-1}\text{L}\right]$	555/105	615/–0.65
			485/+0.10
			315/+1.10
	$\left[{\text{CuH}}_{-2}\text{L}\right]$	550/130	510/–2.50
			315/+1.40
	$\left[{\text{Cu}}_{2}{\text{H}}_{-4}\text{L}\right]$	545/140	590/–1.55
			505/+1.05
			335/–0.90
